# 505 The Successful Development of a Digital Peer Supporter Training Program - How It is Done

**DOI:** 10.1093/jbcr/irac012.136

**Published:** 2022-03-23

**Authors:** Sandra J Cramolini, Jessica Irven, Pam Peterson

**Affiliations:** Phoenix Society for Burn Survivors, Knoxville, Tennessee; Phoenix Society for Burn Survivors, Durham, North Carolina; Phoenix Society for Burn Survivors, Grand Rapids, Michigan

## Abstract

**Introduction:**

The global pandemic of 2020 and 2021 has had an unforgettable impact on our lives and community well-being. Burn survivors are a particularly vulnerable population for social isolation and loneliness and, therefore, an amplified need for social connection. This abstract outlines the strategic structure, curriculum, and training required to move an in-person course to an innovative digital model.

**Methods:**

A group of burn subject matter experts (SMEs), including burn survivors, peer supporters, and course instructors, virtually met to brainstorm program redesign, considering the needs of the burn survivors. Research of topics guided manual and course revisions. Experts recommended a website and digital resource review to streamline organizational data collection and participants’ experiences. A scheduled pilot course and evaluation would provide feedback for changes. SMEs created a short turnaround timeline.

**Results:**

After research, the revised digital manual was condensed from 200 to 50 pages, allowing for hyperlinks, abbreviated resources, and replicated content removal. Five major content areas previously taught in-person were converted to recorded PowerPoint learning modules (April – June 2020). The asynchronous modules totaled 4 hours of self-paced learning assigned to the candidates before a set virtual course. A survey monkey assessment after each module evaluates the learner's base knowledge. SMEs developed a 4-hour virtual class incorporating the modules, manual, and group exercises. A designed pre-course worksheet prepares the candidates for group exercises. Based on their burn injury type or situation (parent to parent, survivors with amputations, death of loved one), candidates practice virtual peer support visits. After the first pilot course, a comprehensive evaluation tool assessed participant satisfaction and confidence, curriculum, instructor expertise, and comfort level navigating the virtual model (August 2020). A website landing page centralized course resources and content location, streamlining participant experience and organizational data collection (March 2021). A 2-hour instructor course (n=10) provided an update on the new structure. The instructors also observe a virtual peer supporter course, participate as an instructor in-training, then co-teach with another instructor before independent course instruction (June 2021).

**Conclusions:**

The digital model has trained burn survivors (n=41) in 6 courses over eight months during a pandemic. Burn survivors have trained together from around the globe to one of the calendared courses improving efficiencies. There are monetary and resource cost savings for burn centers and the organization. The digital manual is easily updated, saving in resource allocation. Peer supporters navigate additional online resources for ongoing self-growth through the embedded website landing page.

